# Antibiotic susceptibility of lactic acid bacteria isolated from artisanal coalho cheese in the Agreste region of Pernambuco, Brazil

**DOI:** 10.1007/s42770-026-01924-w

**Published:** 2026-04-08

**Authors:** Anna Giselle Cavalcanti Vaz Mendes Silva, Karla Sequeira Mendonça, José Erick Galindo Gomes, Lavínia Coeli Santos Valério, Mariane Mendes da Silva, Matheus Oliveira Silveira, Gualberto Segundo Agamez Montalvo, Bruna Maria Salotti de Souza, Vladimir da Mota Silveira Filho, Marcelo Mendonça, Keila Aparecida Moreira

**Affiliations:** 1 Departamento de Morfologia e Fisiologia Animal, Universidade Federal Rural de Pernambuco (UFRPE), Rua Dom Manoel de Medeiros, s/n – Dois Irmãos, Recife, CEP: 52171-900 PE Brasil; 2 Laboratório de Pesquisa em Microbiologia e Imunologia, Universidade Federal do Agreste de Pernambuco (UFAPE), Avenida Bom Pastor, s/n – Boa Vista, Garanhuns, PE CEP: 55292-278 Brasil; 3 Laboratório de Microbiologia, Tecnologia Enzimática e Bioprodutos, Universidade Federal do Agreste de Pernambuco (UFAPE), Avenida Bom Pastor, s/n – Boa Vista, Garanhuns, PE CEP: 55292-278 Brasil; 4 Departamento de Estatística e Matemática Aplicada, Universidade Federal do Ceará (UFC), Avenida Mister Hull, s/n – Pici, Fortaleza, CE 60455-760 Brasil; 5 Departamento de Tecnologia e Inspeção de Produtos de Origem Animal, Universidade Federal de Minas Gerais (UFMG), Avenida Antônio Carlos, nº 6627, Belo Horizonte, MG CEP: 31270-901 Brasil; 6 Departamento de Ciências Biológicas, Universidade de Pernambuco (UPE), Rua Capitão Pedro Rodrigues - São José, Garanhuns, PE CEP: 55294-902 Brasil

**Keywords:** Artisanal coalho cheese, Lactic acid bacteria, Food safety, Antibiotic resistance

## Abstract

Artisanal coalho cheese is a dairy derivative traditionally produced in the Northeast region of Brazil, often using raw milk, which helps preserve its microbiota, especially lactic acid bacteria (LAB). This study aimed to isolate and identify LAB present in artisanal coalho cheeses produced in the Agreste region of Pernambuco, Brazil, and to evaluate the phenotypic and genotypic profiles of antibiotic resistance of these microorganisms. Samples of artisanal coalho cheese were collected from eight cities in the Agreste region. LAB strains were isolated and subjected to Gram staining and catalase assays. Isolates were genotypically identified using the 16 S rRNA gene sequencing technique. Out of 111 LAB isolates, 32 were selected for antibiotic susceptibility studies using 12 different antimicrobials. In addition, seven antimicrobial resistance genes were investigated. The isolates were identified as belonging to the genera *Enterococcus*, *Lactococcus*, *Streptococcus*, *Lacticaseibacillus* and *Lactiplantibacillus*. The highest resistance rates were observed for cefoxitin (75.0%) and gentamicin (71.87%). Some strains, such as QCPE34, QCPE48, QCPE63, QCPE112, QCPE123, and QCPE143 were resistant to only one antibiotic, while strain QCPE40 was resistant to eight antibiotics. Overall, high levels of antibiotic resistance were observed, with 68.75% of strains classified as multidrug-resistant (MDR). Tetracycline and/or erythromycin resistance genes were detected in 16 of the 32 strains tested. This raises an alert regarding food safety for consumers, representing a potential risk to the environment and public health.

## Introduction

Coalho cheese is a traditional food from the Northeast region of Brazil, commonly consumed both locally and throughout the country [1]. This type of cheese holds significant economic and cultural value, particularly for small milk producers, many of whom lack access to processing plants. In northeastern Brazil, coalho cheese is produced mainly in the states of Pernambuco, Ceará, Rio Grande do Norte, and Paraíba [2]. This dairy derivative is considered a semi-hard cheese that is white in colour, slightly salty, acidic, and resistant to melting upon exposure to heat [3]. It is traditionally made using cow or goat milk, either raw in an artisanal manner or with pasteurised milk. In the case of artisanal coalho cheese, it retains its distinctive characteristics, primarily due to the lactic acid bacteria (LAB) present in animal milk. These bacteria enable the cheese to carry potentially probiotic microorganisms, classifying it as a potential functional food [4].

LAB belong to a class of bacteria capable of fermenting carbohydrates such as glucose and producing lactic acid as part of their metabolic process for energy production [5]. In recent years, these microorganisms have gained increasing attention in the food industry due to their probiotic properties and the production of various compounds, including short-chain fatty acids, vitamins, organic acids, acetaldehyde, amino acids, and bioactive peptides. These compounds are used, for instance, to enhance the flavour of fermented products, increase nutrient availability in food, reduce harmful substances, extend shelf life, and contribute to the maintenance of homeostasis and consumer health [6].

Owing to their “Generally Recognized as Safe” (GRAS) status granted by the United States Food and Drug Administration (FDA), LAB have been used as reliable microorganisms to produce fermented foods. However, scientific literature has raised concerns about the resistance of LAB in food to various antibiotics, which poses a risk to food safety and public health [7]. Antibiotic resistance has led to increasingly prolonged hospitalisation and increased mortality rates from infectious diseases [8]. This issue is particularly concerning in developing and low-income countries, where bacterial resistance to antibiotics within the food chain is often neglected [9].

The consumption of foods containing antibiotic-resistant LAB may facilitate the transfer of resistance genes to other bacteria in the human gastrointestinal tract. Direct contact between these microorganisms may promote the exchange of resistance genes between bacterial populations [10, 11]. According to Li et al. [12], evaluating the antibiotic susceptibility and the presence of resistance genes in food-derived LAB is essential for assessing their probiotic capacity.

The investigation of antibiotic susceptibility and antimicrobial resistance genes in LAB isolated from artisanal coalho cheese, a widely consumed product in the Northeast region of Brazil, presents an innovative approach, especially in view of the growing global concerns about antimicrobial resistance. This study aimed to isolate and identify LAB present in artisanal coalho cheeses produced in the Agreste region of Pernambuco, Brazil, and to characterise the phenotypic and genotypic antibiotic resistance profiles of these microorganisms.

## Materials and methods

### Sampling

Lactic acid bacteria were isolated from samples of artisanal coalho cheese samples collected from 30 different farms in eight cities in the Southern Agreste region of Pernambuco, Brazil: Venturosa (-8.57650 S, -36.87577 W) (*n* = 9); Pedra (-8.49913 S, -36.93744 W) (*n* = 8); São Bento do Una (-8.52175 S, -36.44292 W) (*n* = 6); Garanhuns (-8.89071 S, -36.49648 W) (*n* = 2); Paranatama (-8.91806 S, -36.65206 W) (*n* = 2); Sanharó (-8.36153 S, -36.56357 W) (*n* = 1); Capoeiras (-8.73725 S, -36.63310 W) (*n* = 1); and Itaíba (-8.94548, -37.42000 W) (*n* = 1). All samples were collected randomly directly from properties, transported, and stored in isothermal boxes containing recyclable ice. They were then sent to the Laboratory Support Center for Research at the Federal University of Agreste de Pernambuco (CENLAG – UFAPE), to the Microbiology and Immunology (LAPEMI) and Microbiology, Enzymatic Technology and Bioproducts (LMTEB) laboratories, where they were kept under refrigeration (5 °C ± 2 °C) and processed within 24 h of collection.

### Isolation of lactic acid bacteria

LAB were isolated as described by Medeiros et al. [13]. A 25 g sample of the internal portion of each artisanal coalho cheese was processed and aseptically transferred to sterile plastic bags containing 225 mL of 1% (v/v) peptone water, corresponding to a dilution of 10^− 1^. The samples were then homogenised using a stomacher (SpLabor, São Paulo) for 3 min. After homogenization, 1 mL aliquots were transferred to 9 mL of 0.1% (v/v) peptone water, corresponding to a dilution of 10^− 2^, and serial dilutions were performed up to 10^− 6^. A total of 100 µL of each dilution were seeded in Petri dishes by the surface inoculation technique (spread plate), on Man Rogosa and Sharpe (MRS) agar (Difco, Becton Dickinson Co., Sparks, MD, USA), and incubated in anaerobic jars at 30 °C for 72 h.

### Phenotypic characterization of lactic acid bacteria

The characteristic LAB colonies on MRS agar were selected and subjected to phenotypic characterisation using Gram staining and catalase production assays. Gram-positive and catalase-negative isolates were cultured in MRS broth at 37 °C for 24 h/48 hours and stored in cryogenic tubes with 20% (v/v) glycerol at -70 °C. For activation, the strains were inoculated (2%, v/v) in 10 mL of MRS broth and incubated aerobically for 24 h/48 hours at 37 °C.

### Genotypic identification of LAB isolates

Phenotypically characterised LAB were cultured in MRS broth and subjected to genomic DNA extraction using the adapted glass bead method described by Green and Sambrook [14]. Briefly, the bacterial pellet (1 mL) was resuspended in 150 µL of STES lysis buffer (0.2 M Tris-HCl; 0.5 M NaCl; 0.1% SDS; 0.01 M EDTA; pH 7.6), to which approximately 50 µL of glass beads (0.1 mm Glass beads – Sigma-Aldrich) measured in the 1.5 mL tube and 150 µL of phenol-chloroform-isoamyl alcohol (25:24:1; Sigma-Aldrich) were added. After centrifugation at 13,000 x *g* for 5 min, the supernatant was collected and precipitated with absolute alcohol and 3 M sodium acetate for 20 min at -70 °C. A second centrifugation was performed for 20 min at 13,000 x g and the pellet washed with 70% alcohol. The presence and quality of the genomic DNA were verified on a 0.8% agarose gel, stained with SYBR Safe (Invitrogen - Thermo Fisher Scientific), and visualised using a photo documentation system under ultraviolet light.

Genotypic identification was performed using universal bacterial primers for the 16S rRNA gene sequence (27F: 5’-AGAGTTTGATCCTGGCTCAG-3’ and 1492R: 5’-CTACGGCTACCTTGTTACGA-3’) and amplified by the Polymerase Chain Reaction (PCR) technique following the recommendation described by Hou et al. [15]. The reaction mixture included 1 µL of DNA (~ 50 ng/µL), 0.5 µL of each primer and 4 µL of FirePol MasterMix^®^ (Solis Biodyne), completing the volume to 20 µL. PCR amplification was carried out in an Aeris thermocycler (Esco – Lifesciences, Singapore) with the following conditions: initial denaturation at 95 °C for 6 min, followed by 40 cycles of denaturation at 95 °C for 30 s, annealing at 50 °C for 1 min, and extension at 72 °C for 1 min. The amplicons were subjected to 1% agarose gel electrophoresis and stained with SYBR Safe (Invitrogen).

Genetic sequencing for identification at the genus and species levels was performed at the Human Molecular Genetics Laboratory (LGMH), Genetics Department of the Federal University of Pernambuco (UFPE). Sequencing was performed using capillary electrophoretic separation on an ABI 3500 Genetic Analyser (Applied Biosystems).

### Antimicrobial susceptibility testing

The antimicrobial susceptibility profile was performed using the disk diffusion method proposed by Charteris et al. [16], on MRS agar, following the guidelines of the European Committee on Antimicrobial Susceptibility Testing (EUCAST, 2021). A total of twelve antibiotics (CECON – São Paulo, Brazil) were selected based on the list proposed by the European Food Safety Authority [17], which included: aminoglycosides (gentamicin, 120 µg); macrolides (erythromycin, 15 µg); amphenicols (chloramphenicol, 30 µg); tetracyclines (tetracycline, 30 µg); glycopeptides (vancomycin, 30 µg); penicillins (amoxicillin with clavulanate, 30 µg; ampicillin, 10 µg; penicillin, 10 U; and piperacillin with tazobactam, 36 µg); carbapenems (meropenem, 10 µg); cephalosporins (cefoxitin, 30 µg) and quinolones (ciprofloxacin, 5 µg). *Escherichia coli* ATCC 25,922 was used as the control. After incubation at 30 °C for 24 h, the inhibition zones were read using a digital calliper. The susceptibility profile to the antimicrobials used was expressed in millimetres. The isolates were classified as resistant (R), intermediate (I), or sensitive (S) according to the values ​​proposed by Chateris et al. [16] and Toushik et al. [18]. The tested isolates were then grouped based on their categories using categorical coefficients in R software (R Core Team, 2024).

### Identification of multidrug resistance and multiple antibiotic resistance index calculation

The number of isolates that demonstrated resistance to the selected antibiotics was calculated and presented as the antibiotic resistance rate (%). Multidrug resistance (MDR) was defined as resistance to more than two classes of antibiotics. The multiple antibiotic resistance (MAR) index of the isolates was calculated by dividing the number of antibiotics to which the isolate was resistant by the total number of antibiotics to which the isolate was exposed [7].

### Antimicrobial resistance gene research

Genes that may be involved with the resistance to ampicillin, erythromycin, gentamicin, tetracycline, neomycin, streptomycin, and vancomycin were investigated. The primers used in this study are listed in Table 1. Amplification was performed using the FirePol MasterMix^®^ kit (Solis Biodyne) in a total reaction of 20 µL, containing 1 µL of DNA (~ 50 ng), 0.5 µL of each forward and reverse primer, and 4 µL of the mix. The amplification was carried out in an Aeris thermocycler (Esco), using the following conditions: initial denaturation at 94 °C for 10 min; followed by 35 cycles at 94 °C for 30 s; annealing for 1 min at the specific temperature for each primer (55 °C for vanco; 52 °C for tetM; 59.7 °C for neo; 55.4 °C for gent; 55 °C for ermB; 51 °C for amp; 58.6 °C for strept), followed by extension at 72 °C for 1 min, and final elongation at 72 °C for 5 min.

**Table 1:** PCR primers used for the detection of antibiotic resistance genes in LAB strains

Gene/target	Primers	Sequence (5’ to 3’)	Size (bp)	Reference
*van/vancomycin*	Uv1	TTGGCGTCGTGTTAGGGA	503	Wang et al. [46].
	Lv2	CTGATGCTGCGTGGGAATAGG		
*aadB/gentamycin*	Ug1	CACAACGCAGGTCACATTGATA	414	Wang et al. [46].
	Lg2	GGTACTTCATCGGCATA		
*aph/neomycin*	Un1	ACAAGATGGATTGCACGCAGGT	624	Wang et al. [46].
	Ln2	CGGCCACAGTCGATGAAT		
*aadA2/streptomycin*	Ust1	GCTTACCTCGCCCGTTAGACAT	762	Wang et al. [46].
	Lst2	CCAAGTGATCTGCGCGTGA		
*tetM/ tetracycline*	tetM-F	GGTGAACATCATAGACACGC	401	Gad et al.. [48]
	tetM-R	CTTGTTCGAGTTCCAATGC		
*ermB/erythromycin*	ermB-F	CATTTAACGACGAAACTGGC	405	Gad et al.. [48]
	ermB-R	GGAACATCTGTGGTATGGCG		
*bla/β-lactamase*	Bla-F	CAT ART TCC GATAAT ASM GCC	297	Hummel et al. [23]
	Bla-R	CGT STT TAA CTA AGT ATS GY		
16 S rRNA	27-F	AGAGTTTGATCCTGGCTCAG	1465	Hou et al. [15].
	1492-R	GGTTACCTTGTTACGACTT		

### Statistical analysis

The results of the antimicrobial susceptibility testing were expressed as the mean of inhibition zone diameters obtained from triplicates. The isolates were classified as Sensitive, Intermediate, or Resistant based on the specific established breakpoints. To evaluate the similarity of antibiotic resistance profiles among the LAB isolates, Hierarchical Cluster Analysis (HCA) was performed. A heatmap was generated using the ‘pheatmap’ package in R software, with isolates clustered using Euclidean distance and the complete linkage method to group strains based on their resistance classification scores (Sensitive, Intermediate, Resistant).

## Results and discussion

### Phenotypic characterization of lactic acid bacteria

From the collected artisanal coalho cheese samples, 159 distinct colonies with growth characteristics suggestive of LAB were selected and isolated, including relatively small colonies with a round shape, white colour, and delimited edges, as described by Cavalcante e*t al* [19]. In the Gram staining test, 125 (78.61%) of the 159 isolates were Gram-positive, and 111 (88.80%) were catalase-negative. Among the 111 Gram-positive and catalase-negative strains, 107 presented with coccal morphology, 2 with bacilli and 2 were classified as coccobacilli. LAB are gram-positive, catalase-negative microorganisms that can be coccus-shaped, bacilli, or coccobacilli, with single, double, or triple cells and can also form small or large chains [20].

Albayrak and Duran [21] evaluated 40 isolates from samples of white cheese made with unpasteurized milk and without commercial starter cultures in Turkey. They observed that all strains were Gram-positive, catalase-negative, and coccal-shaped. Similarly, Medeiros et al. [13]. isolated 456 LAB strains from 28 artisanal coalho cheese samples produced in Paraíba, Brazil, of which 425 were cocci and 31 were bacilli. According to Moraes et al. [22]., the microbial population of each dairy product varies according to the geographic region where it is produced and can be attributed to variations in the milk used, the predominant climate, and processing methods.

### Identification of lactic acid bacteria

All 111 Gram-positive and catalase-negative isolates were subjected to molecular identification. Sequencing of the 16 S rRNA gene revealed five LAB genera: *Enterococcus*, *Lactococcus*, *Streptococcus*, *Lacticaseibacillus* and *Lactiplantibacillus* (Table 2). A total of 17 species were identified, with the following distribution: *Enterococcus faecalis* (26.12%); *Lactococcus lactis* subsp. *lactis* (24.32%); *Enterococcus durans* (14.41%); *Lactococcus lactis* subsp. *hordniae* (9%); *Streptococcus lutetiensis* (5.4%); *Enterococcus casseliflavus* (3.6%); *Lactococcus petauri* (2.7%); *Streptococcus infantarius* (2.7%); *Enterococcus hirae* (1.8%); *Enterococcus lactis* (1.8%); *Lactococcus cremoris* subsp. *tructae* (1.8%); *Lactococcus garvieae* subsp. *bovis* (1.8%); *Enterococcus faecium* (0.9%); *Lactococcus garvieae* subsp. *garvieae* (0.9%); *Streptococcus gallolyticus* subsp. *macedonicus* (0.9%); *Lacticaseibacillus paracasei* subsp. *tolerans* (0.9%); *Lactiplantibacillus pentosus* (0.9%).

**Table 2:** Identity of LAB isolated from artisanal coalho cheese produced in Agreste region of Pernambuco, Brazil

Isolate code	*Sample Location	Identification by 16 S rDNA
QCPE1	L01	*Enterococcus durans*
QCPE2	L01	*Enterecoccus faecium*
QCPE3	L01	*Lactococcus lactis* subsp. *lactis*
QCPE4	L01	*Enterococcus durans*
QCPE5	L02	*Enterococcus hirae*
QCPE7	L03	*Enterococcus faecalis*
QCPE9	L03	*Lactococcus cremoris* subsp. *tructae*
QCPE10	L03	*Enterococcus lactis*
QCPE11	L03	*Enterococcus lactis*
QCPE12	L03	*Enterococcus durans*
QCPE13	L03	*Enterococcus hirae*
QCPE15	L03	*Enterococcus faecalis*
QCPE16	L03	*Enterococcus durans*
QCPE19	L03	*Lactococcus lactis* subsp. *lactis*
QCPE20	L03	*Enterococcus durans*
QCPE21	L03	*Enterococcus faecalis*
QCPE22	L03	*Enterococcus durans*
QCPE23	L03	*Enterococcus faecalis*
QCPE25	L03	*Enterococcus durans*
QCPE26	L03	*Enterococcus faecalis*
QCPE27	L03	*Enterococcus faecalis*
QCPE28	L03	*Enterococcus faecalis*
QCPE29	L03	*Enterococcus faecalis*
QCPE31	L04	*Enterococcus durans*
QCPE32	L04	*Enterococcus faecalis*
QCPE33	L04	*Enterococcus faecalis*
QCPE34	L04	*Lactococcus lactis* subsp. *lactis*
QCPE35	L04	*Enterococcus faecalis*
QCPE36	L04	*Enterococcus durans*
QCPE37	L04	*Enterococcus faecalis*
QCPE38	L04	*Lactococcus lactis* subsp. *lactis*
QCPE39	L04	*Lactococcus lactis* subsp. *hordniae*
QCPE40	L04	*Lactococcus lactis* subsp. *lactis*
QCPE41	L04	*Lactococcus lactis* subsp. *lactis*
QCPE42	L04	*Enterococcus faecalis*
QCPE43	L04	*Lactococcus lactis* subsp. *lactis*
QCPE44	L04	*Lactococcus lactis* subsp. *lactis*
QCPE45	L04	*Enterococcus faecalis*
QCPE46	L04	*Lactococcus lactis* subsp. *lactis*
QCPE48	L04	*Lactococcus lactis* subsp. *lactis*
QCPE49	L04	*Enterococcus faecalis*
QCPE50	L04	*Enterococcus faecalis*
QCPE54	L05	*Lactococcus lactis* subsp. *lactis*
QCPE55	L05	*Enterococcus faecalis*
QCPE56	L04	*Enterococcus faecalis*
QCPE57	L04	*Lactococcus lactis* subsp. *hordniae*
QCPE58	L04	*Enterococcus faecalis*
QCPE60	L04	*Lactococcus lactis* subsp. *hordniae*
QCPE61	L04	*Lactococcus lactis* subsp. *hordniae*
QCPE62	L02	*Lactococcus cremoris* subsp. *tructae*
QCPE63	L02	*Lactococcus lactis* subsp. *hordniae*
QCPE65	L03	*Enterococcus durans*
QCPE66	L06	*Lactococcus lactis* subsp. *lactis*
QCPE67	L06	*Streptococcus lutetiensis*
QCPE68	L06	*Streptococcus lutetiensis*
QCPE69	L06	*Streptococcus lutetiensis*
QCPE70	L06	*Enterococcus faecalis*
QCPE71	L06	*Lactococcus lactis* subsp. *Lactis*
QCPE77	L04	*Enterococcus faecalis*
QCPE80	L04	*Lactococcus lactis* subsp. *lactis*
QCPE84	L04	*Streptococcus infantarius*
QCPE85	L04	*Streptococcus lutetiensis*
QCPE87	L04	*Lacticaseibacillus paracasei* subsp. *tolerans*
QCPE88	L04	*Lactococcus lactis* subsp. *lactis*
QCPE89	L04	*Enterococcus durans*
QCPE90	L04	*Lactococcus lactis* subsp. *lactis*
QCPE96	L07	*Enterococcus durans*
QCPE97	L07	*Lactococcus lactis* subsp. *lactis*
QCPE98	L07	*Enterococcus faecalis*
QCPE99	L07	*Lactiplantibacillus pentosus*
QCPE100	L07	*Enterococcus faecalis*
QCPE101	L07	*Enterococcus faecalis*
QCPE103	L07	*Enterococcus faecalis*
QCPE105	L07	*Streptococcus lutetiensis*
QCPE109	L03	*Lactococcus lactis* subsp. *lactis*
QCPE111	L03	*Lactococcus lactis* subsp. *lactis*
QCPE112	L03	*Lactococcus lactis* subsp. *hordniae*
QCPE114	L03	*Lactococcus lactis* subsp. *Lactis*
QCPE118	L03	*Enterococcus durans*
QCPE119	L03	*Lactococcus garvieae* subsp. *garvieae*
QCPE121	L03	*Enterococcus casseliflavus*
QCPE122	L07	*Enterococcus casseliflavus*
QCPE123	L07	*Lactococcus garvieae* subsp. *bovis*
QCPE127	L07	*Lactococcus garvieae* subsp. *bovis*
QCPE128	L07	*Enterococcus durans*
QCPE129	L07	*Enterococcus faecalis*
QCPE130	L07	*Lactococcus petauri*
QCPE131	L07	*Lactococcus petauri*
QCPE132	L07	*Enterococcus durans*
QCPE133	L07	*Lactococcus lactis* subsp. *lactis*
QCPE135	L07	*Lactococcus petauri*
QCPE143	L04	*Lactococcus lactis* subsp. *lactis*
QCPE145	L03	*Enterococcus casseliflavus*
QCPE146	L03	*Lactococcus lactis* subsp. *lactis*
QCPE149	L03	*Streptococcus gallolyticus* subsp. *macedonicus*
QCPE152	L07	*Lactococcus lactis* subsp. *lactis*
QCPE153	L07	*Enterococcus casseliflavus*
QCPE156	L03	*Lactococcus lactis* subsp. *hordniae*
QCPE157	L07	*Lactococcus lactis* subsp. *hordniae*
QCPE162	L07	*Streptococcus infantarius*
QCPE163	L07	*Streptococcus lutetiensis*
QCPE166	L03	*Streptococcus infantarius*
QCPE168	L03	*Enterococcus faecalis*
QCPE169	L03	*Enterococcus faecalis*
QCPE170	L03	*Lactococcus lactis* subsp. *hordniae*
QCPE173	L08	*Enterococcus faecalis*
QCPE178	L08	*Lactococcus lactis* subsp. *lactis*
QCPE182	L08	*Lactococcus lactis* subsp. *lactis*
QCPE183	L08	*Enterococcus durans*
QCPE187	L07	*Lactococcus lactis* subsp. *hordniae*
QCPE195	L07	*Lactococcus lactis* subsp. *lactis*

*L01: Sanharó; L02: Garanhuns; L03: Venturosa; L04: São Bento do Una; L05: Capoeiras; L06: Itaíba; L07: Pedra; L08: Paranatama

**Fig. 1:**
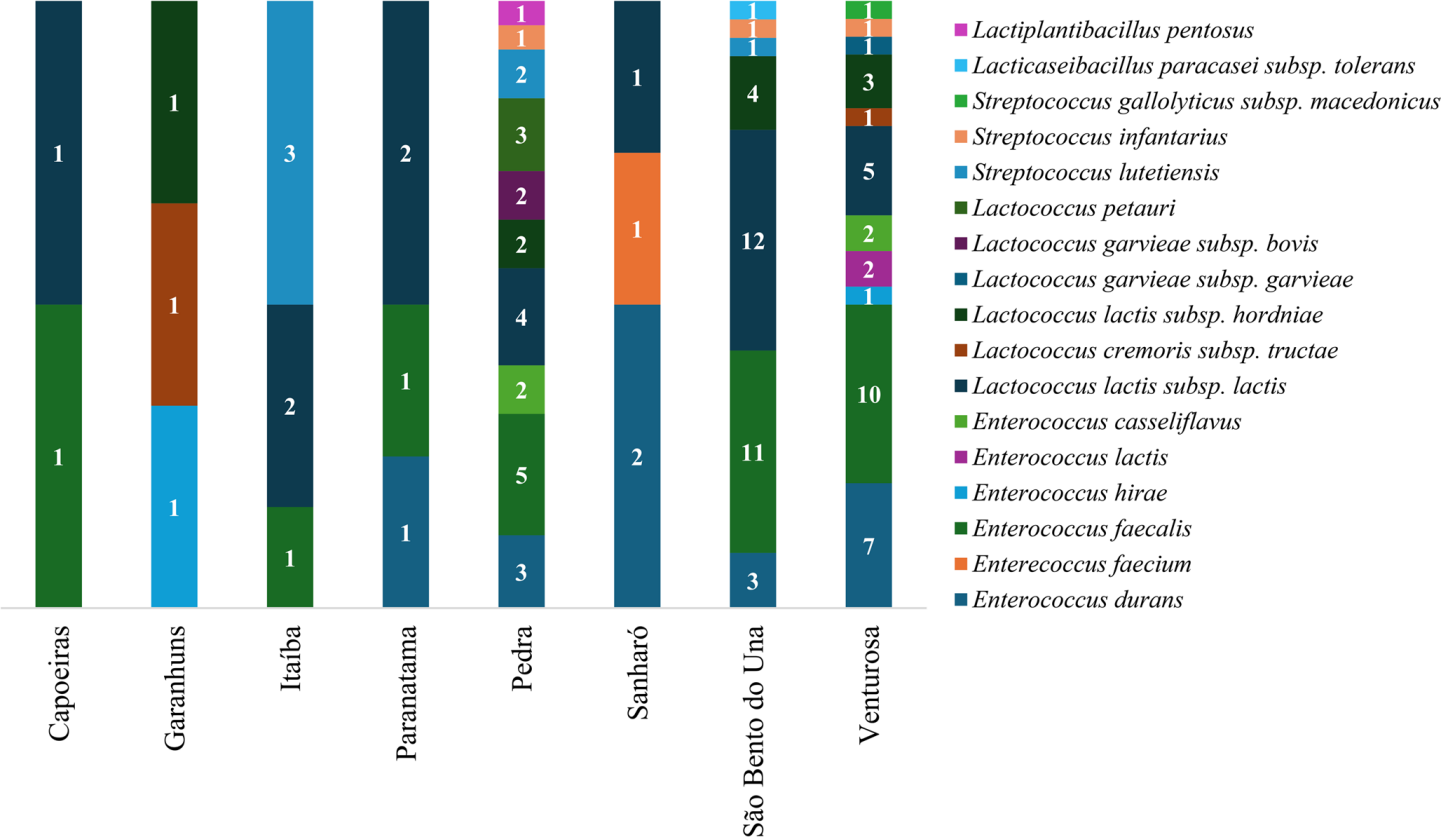
Distribution of LAB isolated from artisanal coalho cheese by cities in the Agreste region of Pernambuco, Brazil

Figure 1 shows the distribution of the different LAB species identified according to the cities where the artisanal coalho cheese samples were obtained. The greatest species diversity was observed in cities with the largest number of processed samples (Venturosa, Pedra, and São Bento do Una). In addition, the city of Capoeiras presented the lowest diversity of LAB species, with only one species of *Lactococcus lactis* subsp. *lactis* and one species of *Enterococcus faecalis* being identified. Notably, *Lactococcus lactis* species were found in samples obtained in all study locations, suggesting that this bacterial genus is important for the organoleptic characteristics of artisanal coalho cheese produced in the Agreste region of Pernambuco.

After genetic identification of the isolates, to obtain a better characterisation of the most prevalent genera with the greatest potential for use in the dairy industry, 32 LAB species from different cities and production sites were selected to assess their antimicrobial susceptibility. These included: *Lactococcus lactis* subsp. *lactis* (*n* = 17), *Lactococcus lactis* subsp. *hordniae* (*n* = 7), *Lactococcus cremoris* subsp. *tructae* (*n* = 2), *Lactococcus garvieae* subsp. *bovis* (*n* = 2), and *Streptococcus lutetiensis* (*n* = 4).

### Antimicrobial susceptibility

Despite their “GRAS” status, LAB strains used in the dairy industry must be free from antibiotic resistance to ensure consumer safety [23, 24]. The FAO/WHO guidelines recommend determining the antibiotic susceptibility of probiotic strains before their consumption or use in food [25].

**Fig. 2:**
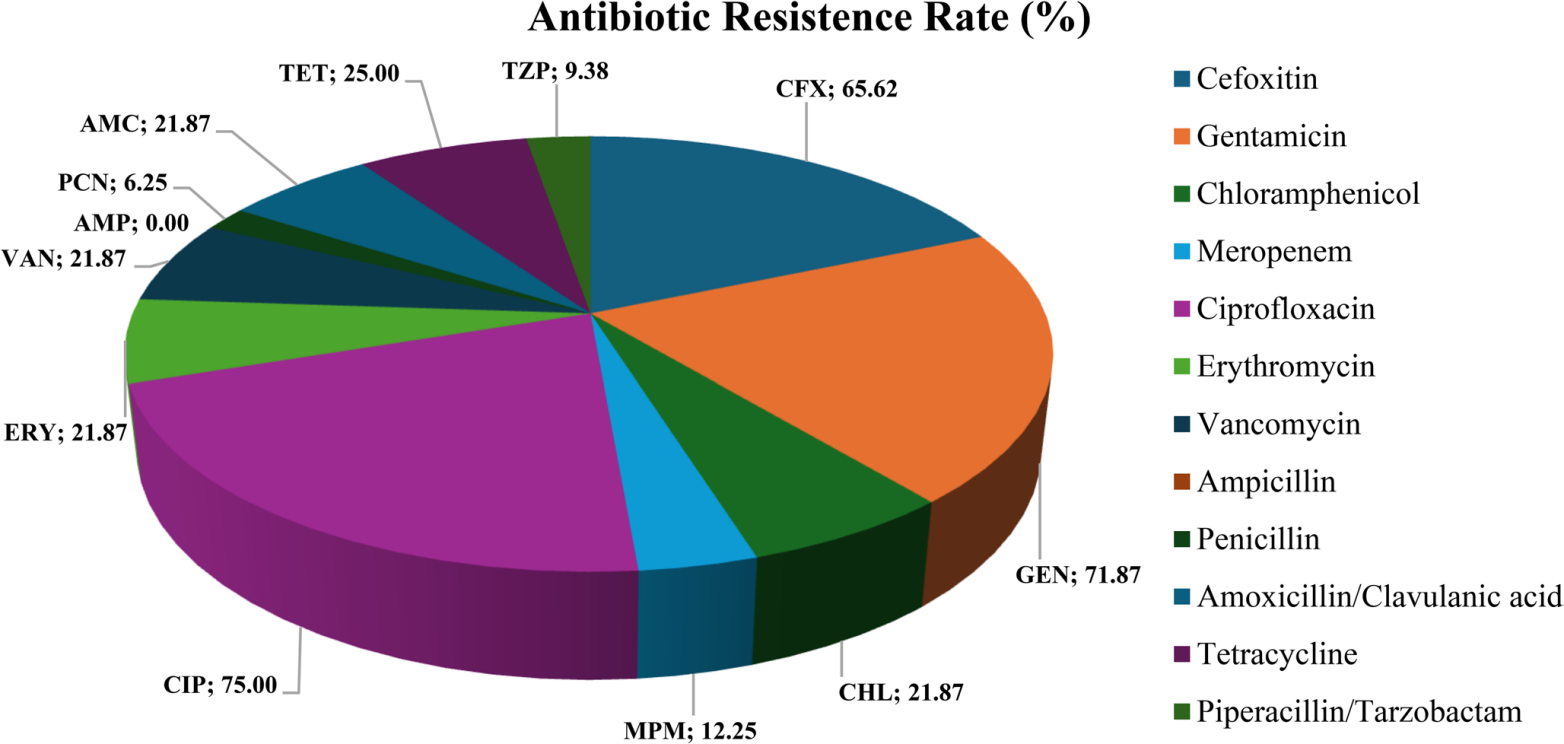
Percentage of antibiotic resistance rate of 32 LAB isolates from artisanal coalho cheese produced in Agreste region of Pernambuco, Brazil

The 32 selected LAB isolates were evaluated for susceptibility to 12 antibiotics, and the results are presented in Fig. 2. The highest resistance was observed against the aminoglycoside gentamicin (71.87%). Among the *Lactococcus* strains, 19 (67.85%) of the 28 isolates showed resistance to this antibiotic. Gentamicin resistance in *Lactococcus* species was also reported [26]. All *Streptococcus* species were resistant to gentamicin. *Streptococcus* possesses an intrinsic resistance to aminoglycosides, which cannot be horizontally transferred, thus posing no risk to human health [27]. Resistance to aminoglycosides such as gentamicin may be related to membrane impermeability, enzymatic inactivation of the antibiotic, or a lack of cytochrome-mediated electron transport, which inhibits drug absorption [7].

Other antibiotics that showed high resistance rates included ciprofloxacin (75%), a quinolone, and cefoxitin (65.62%), a second-generation β-lactam cephalosporin. Ciprofloxacin resistance in *Lactobacillus delbrueckii* subsp. *bulgaricus* (87.1%) and *Streptococcus thermophilus* (79.5%) isolated from fermented products in China has been observed [28]. One study found 100% resistance to cefotaxime (a third-generation cephalosporin) in 23 *Lactococcus* strains isolated from raw cow’s milk [29]. Cephalosporins are a subclass of β-lactam antimicrobials used to treat various types of bacterial infections. Resistance to cephalosporins may result from changes in penicillin-binding proteins (PBPs), alterations in the cell wall structure, or the production of β-lactamase enzymes, which inactivate the antibiotics [30].

**Fig. 3:**
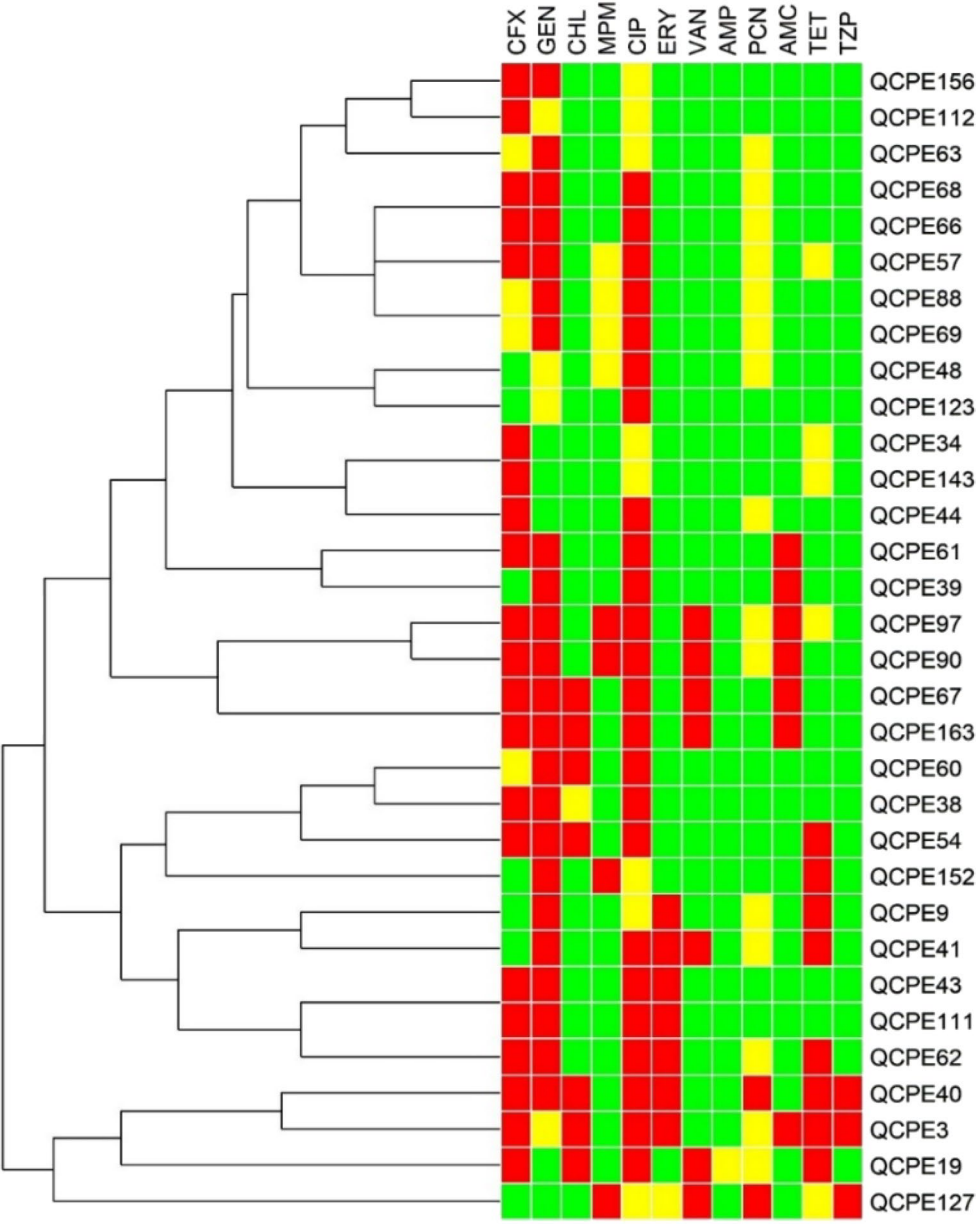
Antibiogram of 32 LAB isolates from artisanal coalho cheese produced in Agreste region of Pernambuco, Brazil, against 12 antibiotics. The panel colors correspond to the type of characteristic, i.e. green for susceptible, yellow for intermediate and red for resistant

According to Mehndiratta and Bhalla [31], an antibiogram is a phenotypic method for differentiating bacterial strains by comparing their susceptibility to different antibiotics. In the antibiogram, isolates with different susceptibilities to antibiotics are considered as different strains. The results, shown in Fig. 3, illustrate the antibiogram, which identified 27 strains among the 32 isolates. For instance, isolates QCPE68-QCPE66, QCPE88-QCPE69, QCPE34-QCPE143, QCPE67-QCPE163 and QCPE43-QCPE111 shared the same antibiotic resistance patterns. However, the QCPE68-QCPE66 and QCPE88-QCPE69 isolate clusters were genetically identified as distinct microorganisms (Table 2). Similarly, the QCPE67-QCPE163 and QCPE43-QCPE111 clusters, although classified as the same species (*Streptococcus lutetiensis* and *Lactococcus lactis* subsp. *lactis*, respectively), were isolated from different cities and thus cannot be considered same strains. The QCPE34-QCPE143 cluster, isolated from the same city but different production sites, contained two *Lactococcus lactis* subsp. *lactis* isolates. Notably, the QCPE34 isolate carried two antibiotic resistance genes, whereas QCPE143 did not, further distinguishing these isolates as distinct strains (Table 3).

**Table 3:** Antimicrobial susceptibility profile of LAB isolated from artisanal coalho cheese produced in Agreste region of Pernambuco, Brazil

Strains		CFX	GEN	CHL	MPM	CIP	ERY	VAN	AMP	PCN	AMC	TET	TZP	MAR Index	Resistance genes
*L. lactis* subsp. *lactis*	QCPE3	R	I	R	S	R	R	S	S	I	R	R	R	0.58	*tet(M)*,* erm(B)*
	QCPE19	R	S	R	S	R	S	R	I	I	S	R	S	0.42	*-*
	QCPE34	R	S	S	S	I	S	S	S	S	S	I	S	0.08	*tet(M)*,* erm(B)*
	QCPE38	R	R	I	S	R	S	S	S	S	S	S	S	0.25	*tet(M)*,* erm(B)*
	QCPE40	R	R	R	S	R	R	S	S	R	S	R	R	0.67	*tet(M)*
	QCPE41	S	R	S	S	R	R	R	S	I	S	R	S	0.42	*tet(M)*
	QCPE43	R	R	S	S	R	R	S	S	S	S	S	S	0.33	*erm(B)*
	QCPE44	R	S	S	S	R	S	S	S	I	S	S	S	0.17	*erm(B)*
	QCPE48	S	I	S	I	R	S	S	S	I	S	S	S	0.08	*-*
	QCPE54	R	R	R	S	R	S	S	S	S	S	R	S	0.42	*-*
	QCPE66	R	R	S	S	R	S	S	S	I	S	S	S	0.25	*-*
	QCPE88	I	R	S	I	R	S	S	S	I	S	S	S	0.17	*-*
	QCPE90	R	R	S	R	R	S	R	S	I	R	S	S	0.50	*-*
	QCPE97	R	R	S	R	R	S	R	S	I	R	I	S	0.50	*-*
	QCPE111	R	R	S	S	R	R	S	S	S	S	S	S	0.33	*-*
	QCPE143	R	S	S	S	I	S	S	S	S	S	I	S	0.08	*-*
	QCPE152	S	R	S	R	I	S	S	S	S	S	R	S	0.25	*-*
*L. lactis* subsp. *hordniae*	QCPE39	S	R	S	S	R	S	S	S	S	R	S	S	0.25	*tet(M)*
	QCPE57	R	R	S	I	R	S	S	S	I	S	I	S	0.25	*tet(M)*
	QCPE60	I	R	R	S	R	S	S	S	S	S	S	S	0.25	*tet(M)*
	QCPE61	R	R	S	S	R	S	S	S	S	R	S	S	0.33	*tet(M)*
	QCPE63	I	R	S	S	I	S	S	S	I	S	S	S	0.08	*-*
	QCPE112	R	I	S	S	I	S	S	S	S	S	S	S	0.08	*-*
	QCPE156	R	R	S	S	I	S	S	S	S	S	S	S	0.17	*-*
*L. cremoris* subsp. *tructae*	QCPE9	S	R	S	S	I	R	S	S	I	S	R	S	0.25	*-*
	QCPE62	R	R	S	S	R	R	S	S	I	S	R	S	0.42	*-*
*L. garvieae* subsp. *bovis*	QCPE123	S	I	S	S	R	S	S	S	S	S	S	S	0.08	*tet(M)*
	QCPE127	S	S	S	R	I	I	R	S	R	S	I	R	0.33	*tet(M)*
*S. lutetiensis*	QCPE67	R	R	R	S	R	S	R	S	S	R	S	S	0.50	*tet(M)*
	QCPE68	R	R	S	S	R	S	S	S	I	S	S	S	0.25	*tet(M)*
	QCPE69	I	R	S	I	R	S	S	S	I	S	S	S	0.17	*tet(M)*
	QCPE163	R	R	R	S	R	S	R	S	S	R	S	S	0.50	*-*

*CFX* Cefoxitin, *GEN* Gentamitin, *CHL* Chloramphenicol, *MPM* Meropenem, *CIP* Ciprofloxacin, *ERY* Erythromycin, *VAN* Vancomycin, *AMP* Ampicillin, *PCN* Penicillin, *AMC* Amoxicillin/clavulanic acid, *TET* Tetracycline, *TZP* Piperacillin/tazobactam

The *Lactococcus lactis* subsp. *lactis* strain QCPE40 exhibited resistance to 66.66% of the tested antibiotics, showing resistance to cefoxitin, piperacillin, gentamicin, chloramphenicol, ciprofloxacin, erythromycin, and tetracycline, as shown in Fig. 3; Table 3. The *Streptococcus lutetiensis* strains QCPE67 and QCPE163 were resistant to 50% of the antibiotics, including cefoxitin, amoxicillin/clavulanate, gentamicin, chloramphenicol, ciprofloxacin, and vancomycin. Uncontrolled exposure to antimicrobial agents imposes selective pressure on bacteria, leading to the development of bacterial strains with high antibiotic resistance [32]. Livestock farming has a high potential as a reservoir of resistant bacteria due to the indiscriminate use of antimicrobials in animal production, especially beta-lactam antibiotics, commonly used in the treatment and prevention of infections [33–35].

Additionally, resistant bacteria can colonise food processing equipment, contributing to cross-resistance and inducing antibiotic resistance against both clinically common and uncommon antimicrobial agents [36]. When a bacterial strain demonstrates greater resistance to a specific antimicrobial compared to other strains of the same taxonomic unit, it is considered to have acquired resistance, necessitating an investigation into the genetic basis of the resistance [18].

In contrast, other antibiotics tested showed low levels of resistance among the evaluated LAB isolates (Fig. 2). Specifically, the *Lactococcus lactis* subsp. *lactis* strains QCPE34, QCPE48, and QCPE143, the *Lactococcus lactis* subsp. *hordniae* strains QCPE63 and QCPE112, and the *Lactococcus garvieae* subsp. *bovis* strain QCPE123 were resistant to only one antibiotic: cefoxitin for QCPE34, QCPE143, and QCPE112; gentamicin for QCPE63; and ciprofloxacin for QCPE48 and QCPE123 (Fig. 3; Table 3). Among the β-lactams, no resistance to ampicillin was observed, while resistance to penicillin was recorded at 6.25%, followed by piperacillin (9.38%), meropenem (12.5%) and amoxicillin/clavulanate (21.87%). For other antibiotic classes, resistance rates were 25% for tetracycline, and 21.87% for vancomycin, erythromycin, and chloramphenicol. Similar results were observed in a study of *Lactobacillus* species isolated from mozzarella cheese, which found no resistance to ampicillin among the LAB evaluated [37]. Margalho et al. [4] evaluated 220 LAB isolates from artisanal cheeses produced in Brazil and observed higher resistance rates for ciprofloxacin (81.4%), gentamicin (94.6%), and vancomycin (97.3%), and all LAB isolates were resistant to streptomycin. However, LAB were sensitive to tetracycline (95%), erythromycin (93.2%), and penicillin (76.4%).

It is important to note that LAB are routinely exposed to various environmental factors during food production, which can drive their adaptation to industrial food processing and storage conditions. This adaptation often involves the synthesis of stress-response proteins, through the induction of antibiotic resistance genes, leading to the development of cross-resistance to various types of stress. Zarzecka et al. [38] observed a reduction in the expression of aminoglycoside resistance genes (gentamicin and kanamycin) when certain starter and protective LAB strains were exposed to low temperatures (< 5 °C). Further reductions in resistance phenotypes were achieved by food preservation strategies, such as the application of high-pressure processing (HPP) and increased NaCl concentrations [39].

**Fig. 4:**
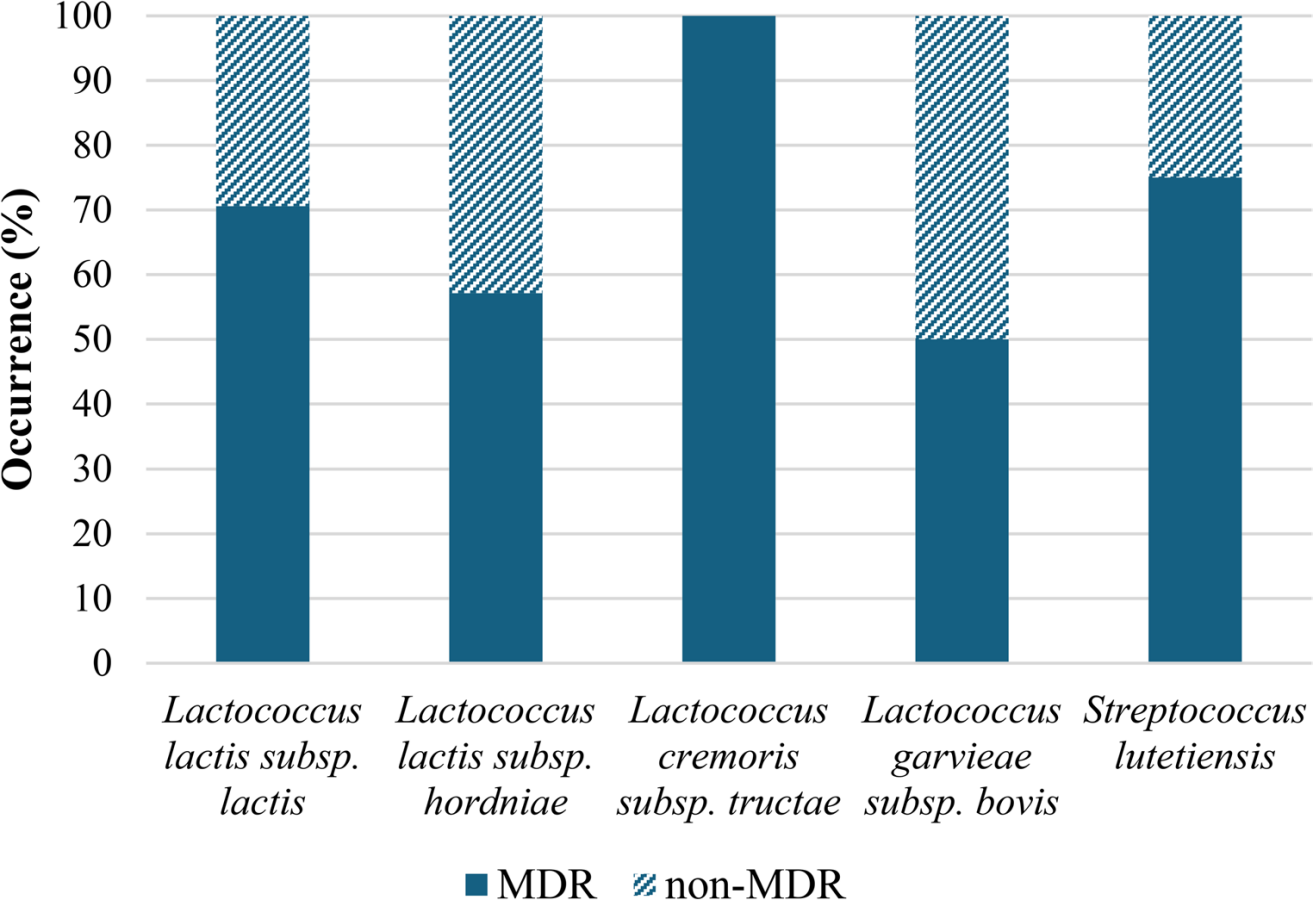
Distributions of MDR and non-MDR strains among 32 LAB isolates from artisanal coalho cheese produced in Agreste region of Pernambuco, Brazil

Moreover, 68.75% (22/32) of the LAB strains in the present study were classified as multidrug-resistant (MDR), as they showed resistance to three or more classes of antibiotics. Of the 17 *Lactococcus lactis* subsp. *lactis* strains evaluated, 12 (70.59%) were MDR (Fig. 4). Additionally, 100% (2/2) of the *Lactococcus cremoris* subsp. *tructae* strains were also MDR. Väkäväinen et al. (2018), isolated 10 MDR LAB strains from LAB in various fermented foods, and Hamdaoui et al. [40], identified MDR strains in *L. lactis* subsp. *lactis* strains isolated from cow milk. Similarly, Ramalho et al. [41], found that *Lactococcus lactis* subsp. *cremoris* LL95 exhibited resistance to amikacin, trimethoprim-sulfamethoxazole, tetracycline, clindamycin, and sulfonamide.

MDR bacteria pose a potential threat to public health, as they can lead to inadequate treatments, resulting in the persistence and spread of infections [42]. While antibiotic-resistant strains are commonly isolated in hospital settings, studies have shown that they are found almost everywhere [43, 44]. LAB can act as reservoirs for antibiotic-resistance genes, which may be transmitted to other bacteria, including those in the human intestinal microbiota, via mechanisms such as plasmids, transposons, or integrons. This gene transfer presents a potential risk to human health [12, 45]. The high prevalence of MDR strains found in the present study may indicate an imminent threat of increasing antimicrobial resistance in Pernambuco, particularly since these LAB were isolated from a widely consumed food in the region. This highlights the potential contribution of food products to the global issue of antibiotic resistance. Therefore, greater attention must be paid to ensure the safe production and consumption of fermented foods.

Table 3 also shows the MAR index for the LAB strains subjected to the antibiogram test. Among the LAB evaluated, 68.75% exhibited a MAR index greater than 0.2, indicating a greater capacity for MDR. According to Ayandele et al. [45], a MAR index greater than 0.2 indicates high exposure to antibiotics, making microorganisms a high-risk source of acquiring resistance. The high MAR index reported here raises an alert for the risks associated with artisanal coalho cheese produced in the Agreste region of Pernambuco, especially given that it is made from raw milk from herds with high antibiotic use, primarily to combat diseases such as mastitis.

In addition to phenotypic resistance, evaluating the presence of resistance genes in the LAB strains isolated in this study is fundamental to understanding the risks inherent in the transfer of these genes to other bacteria. According to Table 3, the strains of *L. lactis* subsp. *lactis* (QCPE40 and QCPE41), *L. lactis* subsp. *hordniae* (QCPE39, QCPE57, QCPE60 and QCPE61), *L. garvieae* subsp. *bovis* (QCPE123 and QCPE127) and *S. lutetiensis* (QCPE67, QCPE68 and QCPE69) showed a positive result for the presence of the tetracycline resistance gene tet(M). While the strains of *L. lactis* subsp. *lactis* QCPE43 and QCPE44 were positive for the gene erm(B). Furthermore, the strains of *L. lactis* subsp. *lactis* QCPE3, QCPE34, and QCPE38 showed positive results for the tet(M) and erm(B) genes. Despite the presence of these resistance genes, there was no phenotypic resistance to tetracycline in strains QCPE39, QCPE57, QCPE60, QCPE61, QCPE123, QCPE127, QCPE67, QCPE68, and QCPE69. Strain QCPE44 not showed phenotypic resistance to erythromycin, and strains QCPE34 and QCPE38, despite the presence of the tet(M) and erm(B) genes, not showed phenotypic resistance to either antibiotic. In this case, the results may indicate that the genes were expressed at very low levels or they are down regulated or could be due to some inactive gene product [46]. According to Ojha et al. [47], the tet(M) gene is responsible for encoding proteins that will confer protection to ribosomes, while the erm(B) gene, resistant to macrolides, encodes the rRNA methylase enzyme that acts on the 23 S ribosomal subunit. Furthermore, according to the authors, these genes are widely found in mobile genetic elements, such as plasmids and transposons, requiring further investigation, along with their transferability through mechanisms of horizontal gene transfer.

## Conclusion

The phenotypic and genotypic methods employed in this study successfully isolated and identified various LAB species from artisanal coalho cheese produced in the Agreste region of Pernambuco, Brazil. The characteristic microbiota of this dairy product is mainly composed of LAB belonging to the genera *Enterococcus* and *Lactococcus*. Moreover, a significant degree of antibiotic resistance was observed among the selected LAB strains, with a high prevalence of MDR strains (22/32) and a high number of strains with an MAR index greater than 0.2 (22/32). Sixteen of the 32 strains evaluated were positive for at least one antibiotic resistance gene. Although the presence of resistance genes does not directly correlate with the antibiotic susceptibility profiles, they represent a potential risk to the environment and public health. Given that LAB are widely used as probiotics, their roles as possible reservoirs for transmissible antibiotic resistance warrants further attention. Furthermore, it is essential that the food industry routinely performs antibiotic susceptibility testing in its production chain to ensure safety and minimise the spread of resistant strains.
